# A Comparison Between Two Bearing Surfaces for Total Hip Arthroplasty—Ceramic-on-Ceramic and Metal–Polycarbonate–Urethane—A Pseudo-Randomized Study

**DOI:** 10.3390/jfb16100371

**Published:** 2025-10-01

**Authors:** Daniel Donaire Hoyas, Eladio Jiménez Mejías, Jesús Moreta, Manuel Sumillera García, Alberto Albert Ullibarri, Jorge Albareda Albareda

**Affiliations:** 1 Servicio Cirugía Ortopédica y Traumatología, Hospital Universitario Poniente, 04700 El Ejido, Spain; albertullibarri@gmail.com; 2 Departamento de Medicina Preventiva y Salud Pública, Facultad de Medicina de la Universidad de Granada, 18016 Granada, Spain; eladiojimenez@ugr.es; 3 Servicio Cirugía Ortopédica y Traumatología, Hospital San Juan de Dios Santurtzi, 48980 Santurce, Spain; chusmoreta2@hotmail.com; 4 Servicio de Cirugía Ortopédica y Traumatología, Hospital Universitario Marqués de Valdecilla, 39008 Santander, Spain; msumi56@gmail.com; 5 Servicio de Cirugía Ortopédica y Traumatología, Hospital Clínico Universitario Lozano Blesa, 50009 Zaragoza, Spain; albaredajorge@gmail.com

**Keywords:** polycarbonate–urethane, ceramic-on-ceramic, total hip arthroplasty, bearing surface, Harris Hip Score, WOMAC, implant survival, patient satisfaction, complications, squeaking

## Abstract

Background: Polycarbonate–urethane (PCU) is a recently developed bearing surface used in prosthetic hip surgery. It offers several theoretical advantages, including an elasticity modulus similar to that of natural cartilage, good lubrication properties, low wear, and the possibility of using large heads. However, comparative clinical experience is limited. The purpose of this study was to analyze the results of the PCU bearing surface and compare them with those of ceramic-on-ceramic (CoC) bearings using the same femoral stem model. (2) Methods: Following a propensity score matching analysis of a prospectively collected database, patients with a primary total hip arthroplasty aged between 18 and 60 years were included. Subjects were divided into two groups (PCU and CoC). Demographic, patient satisfaction, and implant survival data were recorded. Clinical results were evaluated using the Harris Hip Score (HHS) and the Western Ontario and McMaster Universities Osteoarthritis Index (WOMAC). (3) Results: A total of 105 patients were included in each group. All patients exhibited a positive evolution on both the HHS and the WOMAC subscales between pre-op and one year post-op, no statistically significant differences being found between the groups with respect to improvement on the HHS (*p* = 0.172) or the pain (*p* = 0.523), stiffness (*p* = 0.448), and physical function (*p* = 0.255) subscales of the WOMAC. Head sizes in the PCU group were found to be larger, but this was not seen to have any effect on the patients’ clinical status or the prostheses’ dislocation rate. Although the complication rate was similar across the groups (*p* = 0.828), the incidence of squeaking was higher in the PCU group (*p* = 0.010). No differences were observed when comparing the implant survival rate (*p* = 0.427). nor in mean patient satisfaction (*p* = 0.138). (4) Conclusions: No differences were found in terms of clinical results, complications, implant survival, or patient satisfaction between the bearing surfaces under analysis, indicating that all of them are valid alternatives in total hip replacement, although the higher proportion of squeaking observed makes it advisable to exercise some caution.

## 1. Introduction

Tribology has played a major role in the evolution of total hip arthroplasty (THA). Although the metal-on-polyethylene (MoP) bearing surface is supported by a large body of clinical evidence, the degradation of traditional polyethylenes has long been known to result in osteolysis and prosthetic loosening [1,2]. This prompted the adoption of the lower-friction and more wear-resistant ceramic bearing surfaces [3]. However, initial ceramic bearings were not exempt from drawbacks, particularly their high cost and the risk of breakage [4]. The development of polyethylenes and ceramic materials followed a similar evolution, with significant wear reduction rates achieved through the crosslinking of polyethylene chains on the one hand and the introduction of alumina and zirconium-based compounds, which significantly reduced the incidence of fractures, on the other. In fact, the combination of ceramic heads with state-of-the-art polyethylene inserts is currently gaining widespread acceptance [5]. Given that increasingly younger patients are undergoing total hip arthroplasty, there is now a renewed interest in the development of bearing surfaces that may offer lower wear rates, generating biologically inert wear particles [1,3,4,6]. Against this background, one of the most recent innovations has been the introduction of the metal–polycarbonate–urethane (PCU) bearing surface, which offers purported advantages such as an elasticity modulus similar to that of natural cartilage, good lubrication properties, low wear rates, and the possibility of using large heads [6,7,8,9,10,11,12].

Nevertheless, the clinical relevance of those theoretical advantages remains to be determined. Larger femoral heads may reduce the incidence of dislocations and increase the range of motion, which, together with the greater elasticity of inserts, may enhance proprioception and function. In this context, short- and medium-term outcomes are particularly relevant, as they provide the earliest evidence of safety and performance before long-term survival data are available. This is especially important for younger and more active patients, for whom implant longevity is critical and early failures could have major implications for future revision surgery.

The purpose of this study was to analyze the short- and medium-term clinical results of the new PCU bearing and compare them with one of the standard bearings for THA: ceramic-on-ceramic (CoC). The same femoral stem model was used in all cases.

## 2. Materials and Methods

This was a prospective, multicenter cohort study approved by the Centro-Almeria Ethics Committee on 25 February 2015 (approval code 0948-N-14). All patients gave their informed consent before inclusion.

**Figure 1:**
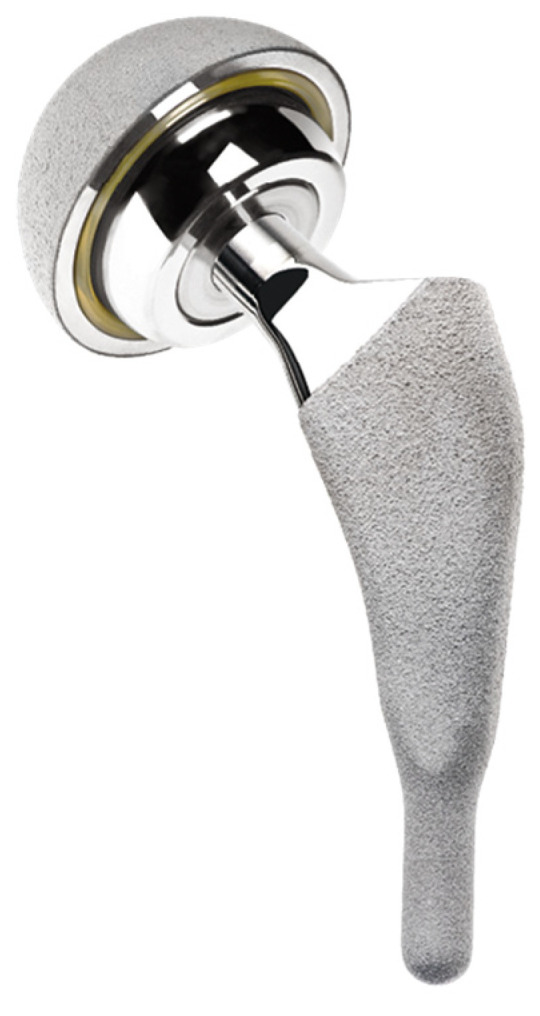
Furlong Evolution femoral stem (JRI Orthopaedics, UK), combined with a polycarbonate–urethane (PCU) liner (TriboFit; JRI Orthopaedics, UK), was used in the study.

The study, carried out in three hospitals with a specialized prosthetic hip surgery unit, included all patients aged between 18 and 60 years who received a primary THA for any reason. Patients who refused to give their informed consent and those who presented with severe bone conditions such as osteomyelitis or bone tumors were excluded from the analysis. Subjects with missing data were not included. All patients received the same femoral stem model (Furlong Evolution, JRI Orthopaedics, Sheffield, UK), with two alternative bearing surfaces: CoC and PCU. The PCU bearing surface used is marketed under the TriboFit brand name (JRI Orthopaedics, Sheffield, UK) (Figure 1). In each case, the choice of bearing surface was dictated by the judgement of the treating surgeon. The surgical approach was determined by the operating surgeon, with anterolateral and posterolateral approaches being the most employed.

**Figure 2:**
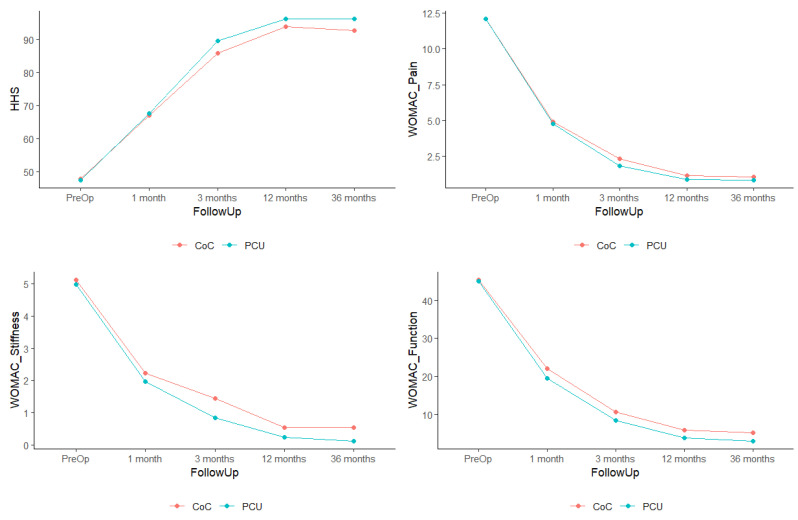
Progression of the patients’ scores showing that their evolution ceases to be clinically and statistically significant from the first year post-op (change between 12 and 36 months; *p* = 1 in all cases).

Apart from demographic data, the study also included an analysis of patient satisfaction, as measured on a scale from 0 to 10. Clinical results were evaluated by means of the Harris Hip Score (HHS) [13] and the three subscales of the Western Ontario and McMaster Universities Osteoarthritis Index (WOMAC) [14]. The data were collected before surgery and at one month, three months, twelve months, three years, and five years postoperatively. The cut-off point selected for the functional analysis was the change observed between the preoperative period and the first 12 months post-op, as a preliminary analysis of the data had shown that patients made no statistically significant improvements beyond that point (Figure 2). This decision is also supported by the previous literature [15,16]. In spite of the foregoing, implant survival and complications were analyzed considering all the patients in the sample from the start of the study to the end of follow-up.

**Figure 3:**
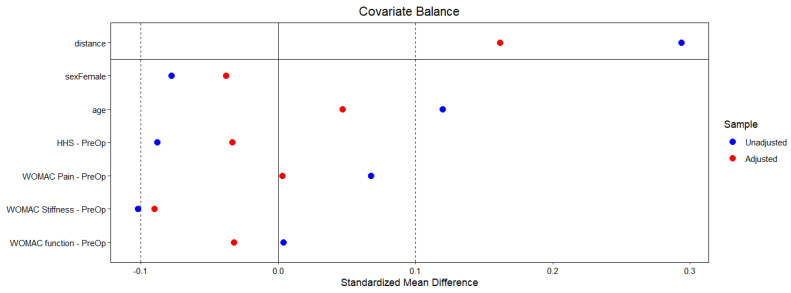
Love plot showing a reduction of the mean difference between variables following application of propensity score matching.

Before the analysis, a propensity score matching (PSM) method was used, employing the optimal matching method with a 1:1 ratio. This approach consolidates multiple covariates (sex, age, and preoperative values of HHS and WOMAC) into a single scalar function, enabling the comparison of subjects with similar characteristics and improving the validity of treatment effect estimates [17,18] (Figure 3).

A descriptive analysis of the data was performed, examining central tendency and dispersion measures. A large sample size made it possible to use parametric tests to compare mean differences. Qualitative variables were analyzed using Pearson’s chi-squared test or the Fisher Exact Test, depending on the magnitude of the expected values. The analysis of the evolution of results over time was performed by means of a repeated measures ANOVA (post hoc comparisons with Bonferroni correction), while implant survival was analyzed with the Kaplan–Meier method. Between-group comparisons were made using the log-rank test. In all cases, statistical significance was set at a *p*-value of 0.05. The analysis of data was conducted with R software (R Development Core Team) v. 4.1.3 [19], and the PSM analysis was carried out with the Match It software [20].

## 3. Results

Following the PSM analysis, a total of 210 patients (148 males/62 females) were included in the study, all of them with at least one year of follow-up. One hundred and five (50%) had received a CoC bearing, and 105 (50%) a PCU bearing surface. The patients’ anthropometric and etiologic characteristics are summarized in Table 1. The groups were homogeneous with respect to sex, mean age, height, and BMI. No statistically significant differences were found between baseline HHS or WOMAC values.

**Figure 4:**
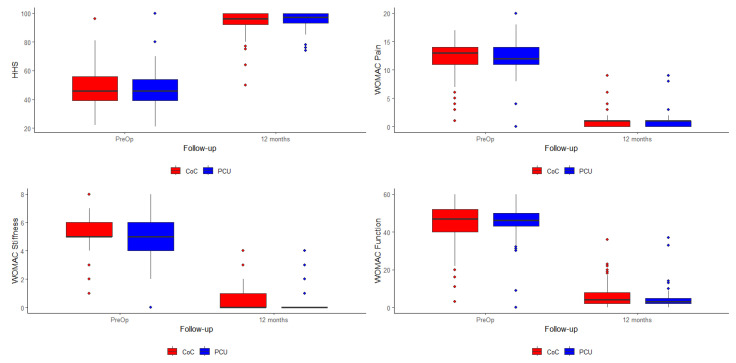
Evolution of the different scoring scales between pre-op and one year post-op.

The data show that although all patients exhibited a positive clinical course from pre-op to one year post-op (*p* < 0.001), no clinically or statistically significant differences were observed in the evolution of the various groups (Table 2 and Figure 4).

As far as the size of the femoral head is concerned, over two-thirds of patients in the PCU group received a large head (above 36 mm), while these heads were present in less than one-third of subjects in the CoC group. The rest received heads equal to or smaller than 36 mm in diameter (Table 3). The difference observed was statistically significant (*p* < 0.001). To evaluate whether femoral head size influenced clinical outcomes, we analyzed head diameter (>36 mm vs. ≤36 mm) as a factor. No significant interaction between head size and time was found for any of the main clinical scores (HHS *p* = 0.937, WOMAC Pain *p* = 0.367, WOMAC Stiffness *p* = 0.368, WOMAC Function *p* = 0.175), indicating that the improvement at 12 months was comparable regardless of femoral head size.

No statistically significant differences were observed when comparing the complication rate of the groups (*p* = 0.828). Moreover, analyzing each complication type separately, statistically significant differences were only found for squeaking, which was more frequent in the PCU group (0.9% vs. 7.6%; *p* = 0.010) (Table 4).

**Figure 5:**
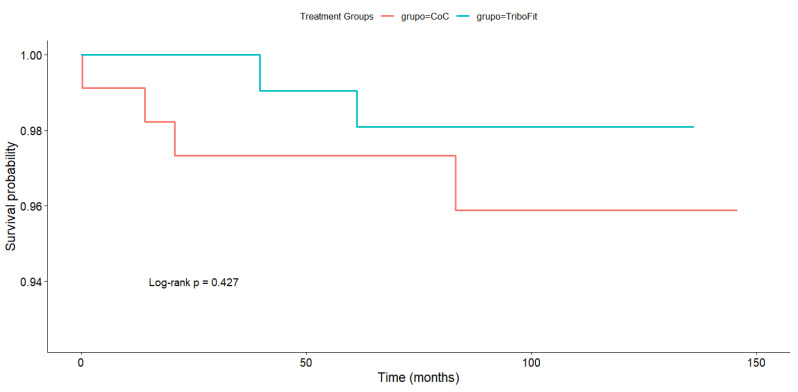
Kaplan–Meier analysis comparing the survival of the two groups.

As regards implant survival (Figure 5), a total of six prosthetic failures were recorded in the series (2.03%), four of which occurred in the CoC group and two in the PCU group. Consequently, taking prosthetic failure for any reason as an endpoint, the 10-year survival rate as estimated using the Kaplan–Meier method was 95.9% in the CoC group and 98.1% in the PCU group. No statistically significant differences were found by the log-rank test between the groups under analysis (*p* = 0.427).

Mean satisfaction (Table 2) was 8.75 ± 1.0 for patients in the CoC group and 8.93 ± 0.8 for those in the PCU group (*p* = 0.138).

## 4. Discussion

The general characteristics of the different bearing surfaces have been widely discussed in the literature [1,3,4,6,21,22], their specific assessment falling outside the scope of the present study. Harder bearings generally result in lower wear rates due to smoother surfaces and greater resistance to deformation. Moreover, such bearings allow the use of larger heads, reducing the risk of dislocation and improving proprioception. However, the virtually complete abandonment of metal-on-metal bearing surfaces because of the severe complications caused by metal ions has left ceramic bearings as the only hard-bearing option [1,5]. CoC bearings stand out for their excellent wear resistance, biocompatible particles, and—in newer designs—improved fracture resistance [3,23]. Their main drawbacks include their high relative cost and the occurrence of squeaking [4], in addition to potential damage to the Morse taper in the event of breakage or revision [3]. Advances in polyethylene manufacturing have also improved its wear performance [3,4], making its use more attractive. The metal–PCU bearing surface, introduced a few years ago, seeks to provide a more natural synovial environment and offers a series of theoretical advantages such as low wear, an elasticity modulus similar to that of natural human cartilage, the possibility to use large heads, a hydrophilic surface, and the generation of bioinert wear particles [7,9,11,24,25,26]. Initial clinical results have been encouraging.

Apart from national implant registries, we are not aware of any previous clinical trial that has compared the clinical results of CoC and PCU. The main finding of the present analysis is the lack of significant differences in function, satisfaction, implant survival, or complications between both groups. Furthermore, our results confirm that patients typically regain physical function within one year of surgery [11].

The present series includes an overly large proportion of young male patients. This is due to the fact that the femoral stem used in all cases was short, typically indicated in patients with good bone quality. However, the use of a validated pseudo-randomization technique ensured group homogeneity in terms of sex, age, height, and BMI. Baseline functional scores were also comparable.

Regarding the analysis of outcome scales, no statistically significant differences were found between the groups on the repeated measures analysis of the HHS and the WOMAC subscales (Table 1 and Table 2).

Only four studies have evaluated the clinical results of the PCU bearing in THA [10,11,12], two of them analyzing the same cohort [10,11]. Mai et al. [11] reported a mean improvement of 46 points on the HHS between the preoperative period and stabilization of the clinical results, which is similar to the 48.6 points achieved by our PCU group. Moroni et al. [10] reported slightly lower improvements (41.5 points), but with mean final values similar to those of our series. Lazic et al. [12], with a larger sample (n = 180) and the same stem model, did not report HHS values, but their descriptive chart suggests similar outcomes to the ones presented here.

Although most studies comparing different bearing surfaces use the HHS to assess functional outcomes [27], comparisons are limited by patient heterogeneity. Robertson et al. [28] analyzed 68 patients under 55 years—similar to our cohort—and reported a final HHS of 95.9, close to our mean of 95.2, though baseline values were not provided.

The WOMAC subscales make it possible to analyze the different aspects associated with the performance of an implant. As was the case with the HHS, our data did not reveal clinically or statistically meaningful differences between the groups on the WOMAC subscales.

Regarding implant survival and complications, the groups demonstrated similar or even better results than those of other series and official registries [29], with estimated survival rates of 95.9% in the CoC group and 98.1% in the PCU group.

Although complication rates were similar, squeaking was more frequent in the PCU group (nine cases vs. one in CoC, *p* = 0.010). These sounds were activity- or time-specific (e.g., morning) and often diminished or disappeared within a year. Previous publications have inconsistently reported squeaking. Moroni et al. [10] and Mai et al. [11] did not mention the occurrence of squeaking. But Sieber et al. [11] noted one subject (2%) with occasional squeaking, and Lazic et al. [12] reported squeaking in up to 12 (6.67%) of their patients. In line with our analysis, Lazic et al. only observed squeaking during the performance of certain activities or at specific times of day, although they do not mention whether squeaking disappears with time. We share Lazic et al.’s prudent stance, as it is not known whether contact stresses can at times be severe enough to damage the surfaces of the PCU bearing. Unfortunately, we were unable to identify a consistent pattern linking this phenomenon to mechanical factors such as implant positioning, head size, or material properties. This suggests that the etiology may be multifactorial and warrants further investigation. It is worth noting that squeaking is not uncommon with other bearing surfaces. In a study based on national registers [2], it was found that 27% of patients with a CoC bearing, 29% of patients with an MoM bearing, and 12% of patients with an MoP bearing experienced squeaking originating in their THA.

One of the advantages of modern ceramic bearings is that they can be used with large femoral heads. In this regard, our data shows that PCU bearings can accommodate even larger heads, given the thin inserts used [6,7,8,9,10,11,12]. Indeed, femoral heads in over two-thirds of patients in our PCU group were over 36 mm in diameter, while this proportion fell to less than one-third in patients in the CoC group (*p* < 0.001). According to most registries, the most common head size in THA is currently 32 mm [30]. Although the mean head size in all the groups in our study was above that figure, we did not observe any differences in clinical outcomes, as no score was significantly influenced by head size. Although large heads are often promoted for improving the range of motion, reducing dislocation risk, and enhancing proprioception [30,31,32,33,34,35]. They have also been linked to increased wear and complications such as trunnionosis [18]. Moreover, benefits appear to plateau beyond 36 mm, where success depends more on the patient’s soft tissue and bone stock quality than on the implant itself [31,32,33,34,35]. Interestingly, all dislocations (N = 3) occurred with large heads, although it must be borne in mind that there are multiple variables capable of impacting the range of motion and dislocation rates [29,30,31,33,34,35,36,37]. This analysis, however, falls outside the scope of this study.

This study is not without limitations. Firstly, we did not include a radiographic analysis of results, as we gave preference to evaluating patient function and implant survival. However, we acknowledge that imaging is crucial for detecting early signs of osteolysis, loosening, or wear that may not yet be clinically evident. Including radiographic assessments in future follow-ups would allow a more comprehensive evaluation of the implant’s long-term performance. Mention should also be made of the limitations inherent in observational studies, such as the absence of randomization and the associated risk of a selection bias. Additionally, the choice between PCU and CoC bearings was left to the surgeon’s discretion, which may introduce allocation bias. While this reflects real-world practice, it should be acknowledged as a limitation that could influence comparability between groups. However, we believe that the large size of the sample analyzed and, particularly, the prospective nature of the study make our findings generalizable, minimizing the potential for a selection or classification bias. Another limitation is the usage of a non-validated 0–10 satisfaction numeric scale. However, its straightforward interpretability and practical utility support its inclusion, despite limitations in cross-study comparability. Although all comparisons were made at one year post-op for the methodological reasons explained above [15,16], mean patient follow-up was 5.62 years (range: 1–10), which confers a measure of robustness to our analysis of implant survival and complications. Nevertheless, we acknowledge that our follow-up limits the ability to fully assess long-term outcomes, particularly regarding the durability of newer materials such as PCU. Extended follow-up, especially focusing on issues like squeaking and wear, will be essential to provide a more comprehensive evaluation of these bearing surfaces.

## 5. Conclusions

No relevant differences were found at the one-year mark between the clinical results obtained with the CoC and PCU bearing surfaces. The complications and survival rates of the different bearings were also similar, as was patient satisfaction. For those reasons, it can be said that the two types of bearing surface are a valid option for THA, although the higher incidence of squeaking observed warrants a certain amount of caution.

## Figures and Tables

**Table 1:** Preoperative situation.

	CoC	PCU	*p*-Value
Demographic Data			
N	105 (50%)	105 (50%)	*-*
Height (cm)	171.0 ± 7.8	172.5 ± 9.4	0.230
BMI (m/kg^2^)	27.4 ± 4	28.5 ± 4	0.053
Sex (M/F)	72/33	76/29	0.545
Age (years)	49.5± 8.7	49.9 ± 8.4	0.739
Diagnosis			
Primary hip arthritis	41 (39.0%)	44 (41.9%)	0.360
Secondary hip arthritis	33 (31.4%)	27 (25.7%)	
Avascular necrosis	24 (22.9%)	27 (27.5%)	
Fractures or sequelae	3 (2.9%)	1 (1.0%)	
Arthritis	2 (1.9%)	0 (0.0%)	
Unknown	2 (1.9%)	6 (5.7%)	
Scores			
HHS—Pre-Op	48.0	47.6	0.802
WOMAC Pain—Pre-Op	12.1	12.1	0.983
WOMAC Stiffness—Pre-Op	5.12	5.00	0.466
WOMAC Function—Pre-Op	45.4	45.0	0.815

**Table 2:** Evolution at one-year follow-up.

	CoC	PCU	*p*-Value
Satisfaction (1 year)	8.75 ± 1.0	8.93 ± 0.8	0.138
Scores			
HHS—Pre-Op	48.0	47.6	0.172
HHS—12 Months	94.0	96.2	
WOMAC Pain—Pre-Op	12.1	12.1	0.523
WOMAC Pain—12 Months	1.1	0.8	
WOMAC Stiffness—Pre-Op	5.12	5.00	0.448
WOMAC Stiffness—12 Months	0.5	0.2	
WOMAC Function—Pre-Op	45.4	45.0	0.255
WOMAC Function—12 Months	5.9	3.8	

**Table 3:** Head diameters.

Head Diameter	CoC		PCU	
	N	%	N	%
Small heads (up to 36 mm)	78	74.3%	29	29.9%
Large heads (over 36 mm)	27	25.7%	68	70.1%

**Table 4:** Complications.

Complication	CoC	PCU	*p*-Value
Cup loosening	1	0	1
Deep infection	2	0	0.497
Dislocation	1	0	1
Periprosthetic fracture	3	0	0.246
Heterotopic ossification	1	0	1
Seroma	2	0	0.497
Squeaking	1	9	0.010 *
Stem loosening	1	1	1
Superficial infection	1	1	1

* Statistically significant differences between the groups.

## Data Availability

The original contributions presented in the study are included in the article; further inquiries can be directed to the corresponding author.

## Abbreviations

The following abbreviations are used in this manuscript:

THA	Total Hip Arthroplasty
MoP	Metal-on-Polyethylene
PCU	Metal–Polycarbonate–Urethane
CoC	Ceramic-on-Ceramic
HHS	Harris Hip Score
WOMAC	Western Ontario and McMaster Universities Osteoarthritis Index
PSM	Propensity Score Matching
